# Mucopolysaccharidosis type I: molecular characteristics of two novel alpha-L-iduronidase mutations in Tunisian patients

**DOI:** 10.1186/1746-1596-6-47

**Published:** 2011-06-03

**Authors:** Latifa Chkioua, Souhir Khedhiri, Hadhami Ben Turkia, Rémy Tcheng, Roseline Froissart, Henda Chahed, Salima Ferchichi, Marie Françoise Ben Dridi, Christine Vianey-Saban, Sandrine Laradi, Abdelhedi Miled

**Affiliations:** 1 Laboratory of Biochemistry, Farhat Hached Hospital, 4000 Sousse - Tunisia; 2 Laboratory of Molecular Biology, University of Pharmacy, 5000 Monastir - Tunisia; 3 Laboratory of Pediatric LaRabta Hospital Tunis-Tunisia; 4 Hereditary Metabolic Diseases Service, Center for Biology and Pathology, Est Hospices Civils Lyon, 69677 BRON CEDEX France

## Abstract

**Abstract:**

**Background**

Mucopolysaccharidosis type I (MPS I) is an autosomal storage disease resulting from defective activity of the enzyme α-L-iduronidase (IDUA). This glycosidase is involved in the degradation of heparan sulfate and dermatan sulfate. MPS I has severe and milder phenotypic subtypes.

Aim of study: This study was carried out on six newly collected MPS I patients recruited from many regions of Tunisia.

Patients and methods: Mutational analysis of the IDUA gene in unrelated MPS I families was performed by sequencing the exons and intron-exon junctions of IDUA gene.

**Results**

Two novel IDUA mutations, p.L530fs (1587_1588 insGC) in exon 11 and p.F177S in exon 5 and two previously reported mutations p.P533R and p.Y581X were detected. The patient in family 1 who has the Hurler phenotype was homozygous for the previously described nonsense mutation p.Y581X.

The patient in family 2 who also has the Hurler phenotype was homozygous for the novel missense mutation p.F177S. The three patients in families 3, 5 and 6 were homozygous for the p.P533R mutation. The patient in family 4 was homozygous for the novel small insertion 1587_1588 insGC. In addition, eighteen known and one unknown IDUA polymorphisms were identified.

**Conclusion**

The identification of these mutations should facilitate prenatal diagnosis and counseling for MPS I in Tunisia.

**Background:**

Mucopolysaccharidosis type I (MPS I) is an autosomal recessive lysosomal storage disorder caused by the deficient activity of the enzyme of α-L-iduronidase (IDUA, EC 3.2.1.76). This glycosidase is involved in the degradation of heparan sulfate and dermatan sulfate. The clinical phenotype of MPS I ranges from the very severe in Hurler syndrome (MPS IH) to the relatively benign in Scheie syndrome (MPS IS), with an intermediate phenotype designated Hurler/Scheie (MPS IH/S) [1]. Isolation of complementary and genomic DNAs encoding human α -L- iduronidase [2,3] have enable the identification of mutations underlying the enzyme defect and resulting in MPS I clinical phenotype. More than 100 mutations have been reported in patients with the MPS I subtypes (Human Gene Mutation Database; http://www.hgmd.org). High prevalence of the common mutations p.W402X and p.Q70X has been described; both of them in the severe clinical forms [4,5]. A high prevalence of common mutation p.P533R has also been described in MPS I patients with various phenotypes [5,6]. In addition, rare mutations including single base substitution, deletion, insertion and splicing site mutation have been identified [7], indicating a high degree of allelic heterogeneity in IDUA gene.

Here, we described two novel IDUA mutations in MPS I Tunisian patients. These lesions were homoallelic in all the patients of the six families investigated as consanguineous marriages are still frequent in Tunisia [8].

## Patients and Methods

### Patients

**Figure 1:**
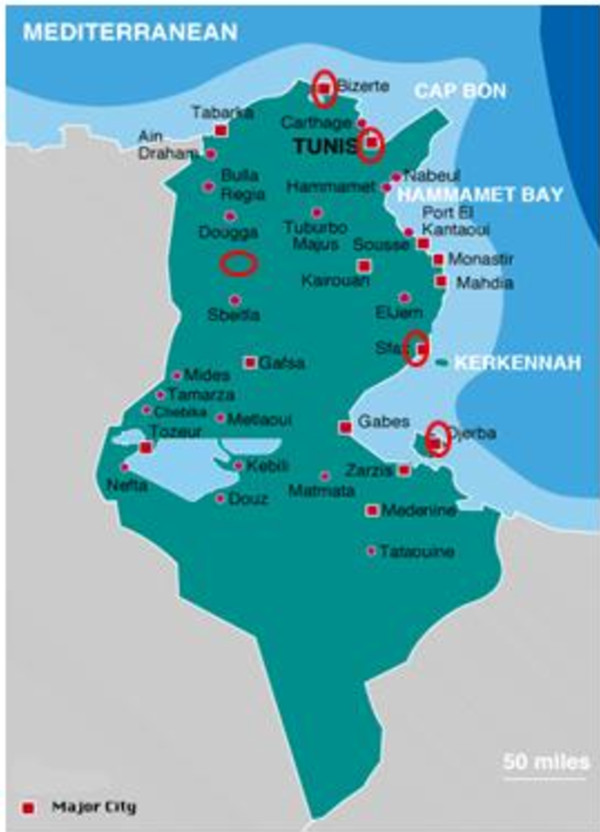
**Regional distribution of studied MPS I patients in Tunisia**.

Six Tunisian unrelated families with MPS I were investigated. The MPS I patients were diagnosed in paediatric clinics of hospital of northern and southern Tunisia (Figure 1). The patients were all offspring of consanguineous marriages between first, second or third cousins. This study was approved by the Ethics committees of the Tunisian hospitals, and all families gave informed consent. Blood samples were collected from the six patients, their parents, and unaffected siblings and relatives. The clinical diagnoses were confirmed by enzymatic assays (Table 1).

**Table 1:** Laboratory and clinical findings of the six MPS I patients

Families	Consanguinity	Tunisian origin	Sex	Age of diagnosis	Age	Leukocyte IDUA activity (% normal)	Mutations identified	Polymorphisms sequences variants
Family 1 Patient 1	3^rd ^cousins	Makthar	F	2 years 5 days	9 years	0.1%	p.Y581X	No polymorphisms

Family 2 Patient 2	2^nd ^cousins	Tunis	F	1 year 6 months	11 years (died)	0.1%	p.F177S	No polymorphisms

Family 3 Patient 3	3^rd ^cousins	Sfax	M	2 years	12 years	0.2%	p.P533R	No polymorphisms

Family 4 Patient 4	1^st ^cousins	Bizerte	M	1 year 8 months	3 years (died)	< 0.1%	p.L530fs (1587_1588inGC)	p.H33Q (c.99 T > G), p.R105Q (c.314G > A), p.N181N (c.543 T > C), IVS5-45 (c.590-45) G > C), IVS5-8 (c.590-8C > T), p.A314A (c.942G > C), IVS7+48(c.972+48A > G), IVS745(c.973- 45G > C), p.A361T (c.1081G > A), p.T388T (c.1164G > C), p.V454I (c.1360G > A), IVS9+36 (c.1402+36T > C), p.R489R (c.1467C > T), IVS12+72 (c.1727T > G)

Family 5 Patient 5	3^rd ^cousins	Djerba Island	M	1year 4 months	4 years	0.2%	p.P533R	No polymorphisms

Family 6 Patient 6	2^nd ^cousins	Djerba Island	M	1 year	2 years	0.3%	p.P533R	p.A8A (c.24 A > C, p.A20A (c.60G > A); p.Q433Q (c.99T > G), IVS2-44 (c.300 44C > T); p.R105Q (c.314G > A); p.N181N (c.543 T > C), IVS5-45 (c.590-45G > C); IVS5-8 (c.590- 8C > T); p.A314A (c.942G > C); IVS7+48 (c.972+48A > G), IVS7-45 (c.973-45 G > C; p.A361 (Tc.1081G > A); p.T388T (c.116G > C); p.V454I (c.1360G > A); IVS9+36 (c.1402+36T > C), p.R489R (c.1467C > T), IVS12+72 (c.1727T > G), 3'UTR+44 (c.1962+44G > C).

### IDUA activity

IDUA activity was determined in sonicated leukocyte pellets as described using the artificial substrate 4-methylumbellifery-α-L-iduronide [9].

### IDUA mutation analysis

Genomic DNA was isolated from venous blood by the phenol/chloroform procedure. Each of the 14 exons and flanking intron-exon junctions of the IDUA gene were amplified from genomic DNAs from the patients and their parents as described previously [10].

Polymerase chain reaction (PCR) was carried out in a volume of 50 μl containing 100 ng genomic DNA, 0.2 mmol/L dNTPs, 0.4 μM of each primer, 1.5 mmol/L Mgcl2, 10% (v/v) DMSO, and 0.15 μl (0.75 units) of Taq Qbiogen (Promega).

## Results

**Figure 2:**
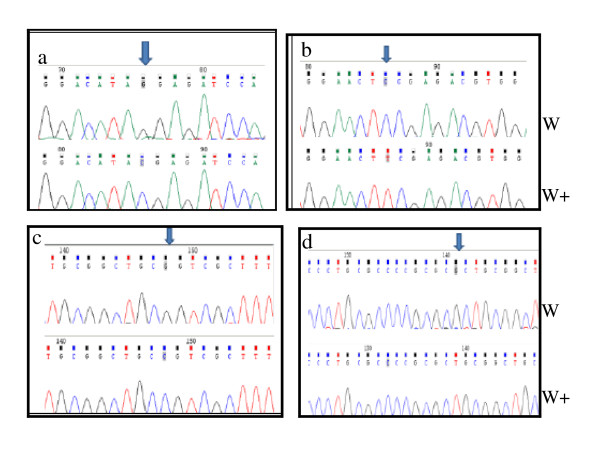
**Direct sequencing of IDUA gene in Tunisian MPS I patients**. 2a: Patient 1 (family 1): p.Y581X point mutation. 2b: Patient 2 (family 2): p.F177S point mutation. 2c: Patients 3, 5 and 6 (families 3, 5 and 6): p.P533R point mutation. 2d: Patient 4 (family 4): p.L530fs frame shift mutation. (w): mutant sequence, (w+): wild -type sequence.

The affected probands in the six families had the typical Hurler phenotype. Two novel mutations, p.L530fs (1587_1588 inGC) in exon 11, and p.F177S in exon 5, and two previously reported mutations, p.P533R and p.Y581X, were detected in the affected patients and their respective parents from the six MPS I families (Table 2). The proband of family 1 with the Hurler phenotype was homozygous for the previously reported p.Y581X nonsense mutation (Figure 2a). The affected MPS IH patient in family 2 was homozygous for the novel p.F177S missense mutation. This mutation was due to a thymine -to- cytosine transversion in codon 177, resulting in the substitution of a polar phenylalanine to an uncharged polar serine residue (Figure 2b). The three affected siblings in families 3, 5 and 6 were homozygous for the previously reported p.P533R missense mutation (Figure 2c). The proband of family 4 was homozygous for a novel small insertion mutation 1587_1588 inGC (Figure 2d). This novel framshift (Leu530fs) in exon 11 changed codon 530 for leucine (CTG) to a premature termination codon (TGA), 30 amino acids downstream from the termination codon of the enzyme glycopeptide. In addition to these mutations, eighteen previously identified and one novel polymorphisms were identified in the Tunisian MPS I alleles (Table 2) [2,7,11,12].

**Table 2:** MPS I mutations in Tunisian patients

Mutations	Nucleiotide change	Base (cDNA)^a^	Exon	Codon	Predicted protein change
p.F177S p.L530fs p.P533R p.Y581X	T > C 1587_1588insGC C > G C > G	530 1587 1598 1743	5 11 11 13	177 530 533 581	Phenylalanine to serine Frame- shift, premature stop Proline to Arginine Tyrosine to stop

^a ^c DNA sequence from NCBI (IDUA NM-000203) with+1 being the A of the ATG initiation codon (n88).

The previously reported polymorphisms were detected in families 4 and 6, including: p.A8A (c.24 A > C), p.A20A (c.60G > A); p.H33Q (c.99 T > G), p.Q433Q (c.99T > G), IVS2-44 (c.300-44C > T); p.R105Q (c.314G > A); p.N181N (c.543 T > C), IVS5-45 (c.590-45G > C); IVS5-8 (c.590-8C > T); p.A314A (c.942G > C); IVS7+48 (c.972+48A > G), IVS7-45 (c.973-45 G > C; p.A361 (c.1081G > A; p.T388T (c.116 G > C); p.V454I (c.1360G > A); IVS9+36 (c.1402+36T > C), p.R489R (c.1467C > T), IVS12+72 (c.1727T > G), and one previously unreported polymorphism, 3'UTR+44 (c.1962+44G > C).

## Discussion

MPS I is the most common mucopolysaccharidosis worldwide, with an average incidence of about 1.7 in 100.000 live births for the severe and mild forms [13]. The incidence of MPS I in Tunisia is also high, estimated at 0.63 in 100.000 live births [14], owing to the high rate of consanguinity [15].

To date, over 100 mutations in the IDUA gene have been described in patients with the MPS I subtypes (Human Gen Database, http://www.hgmd.org).

The molecular analysis of the six newly collected patients in Tunisian families allowed the identification of four mutations including a small insertion (1587_1588 inGC), a novel missense mutation (p.F177S) and two previously reported mutations, p.P533R and p.Y581X (Table 2). Of note, the common Caucasian *IDUA *mutations, p.Q70X and p.W402X, were not present [7].

Patient 1 from family 1 was homozygous for the previously reported p.Y581X mutation in exon 13 of the IDUA gene. The p.Y518X mutation was first described in one patient of Croatian origin. This patient was heteroallelic for the p.Y581X mutation and the Ile583delC mutation [16].

A patient 2 from family 2 was homozygous for the novel p.F177S mutation. This mutation causes a sever instability or loss of IDUA protein function, since the 177 residue was predicted to form the strands of the β barrel with the catalytic residues perched on the C terminal ends of the β strands [17]. The predicted IDUA active site showed different residues, among which the residue Glu178 which is near the Phe177. This mutation presumably resulted from slipped mispairing and repairing during replication. Our study identified the p.177S mutation in patient which had the severe phenotype and undetectable IDUA activity. This concept is consistent with the fact that the phenylalanine at IDUA residue 177 is highly conserved in evolution.

A patient four from family four was homozygous for the novel 1587_1588 inGC mutation that leads to the lack of 30 aminoacid at the amino terminus of the IDUA protein (p.Leu530fs).

The insertion (GC) occurred between two direct repeats (GC) that were separated by only three bases (CCC). The patient 4 did not have detectable IDUA activity and presented with the severe Hurler phenotype.

Patients 3, 5 and 6 from families 3, 5 and 6 respectively were homozygous for the p.P533R mutation. These patients had undectable IDUA activity and the typical Hurler phenotype. The p.P533R missense mutation in exon 11 resulted in a non conservative substitution of a neutral proline for a basic arginine.

The IDUA model begins at residue 36 of the linear sequence and terminates at residue 522, as this is the longest section that can be reliably predicted on the basis of the crystal structure of of the β-D-xylosidase from *Thermoanaerobacterium saccharolyticum *(XyTS, EC 3.2.1.37) [18]. As a result, residues 523-653 do not appear in the IDUA model, consistent with the finding that a proline residue in codon 533 is incapable of forming the main chain hydrogen bonds. A non conservative substitution of a neutral proline for a basic arginie could be predicted to drastically change the orientation of the secondary structure in IDUA protein leading to a severe disease phenotype.

The basis of such regional distribution of p.P533R mutation is not clear. This mutation has been identified in 92% of mutant alleles in 13 MPS I patients from Marroco [6], in 11% of mutant alleles of 27 MPS I patients from Sicily [19], in 62.5% of mutant alleles in 10 MPS I patients from Tunisia [20] and not identified in 3 MPS I patients from Egypt [21], suggesting that the p.P5323R allele possibly originated from a common founder from the Islamic occupation of Sicily [22].

A large number of polymorphisms and non pathogenic sequence variants have been described in the *IDUA *gene [7,23,24] (Table 2). The effect of these sequences variants on the IDUA activity has not been clearly defined especially when they are associated with specific mutations causing the Hurler/Scheie and Scheie phenotypes [7]. The effect of the noncoding [IVS2-44 (c.300-44C > T);IVS5-45 (c.590-45G > C); IVS5-8 (c.590-8C > T);IVS7+48 (c.972+48A > G), IVS7-45 (c.973-45 G > C; IVS9+36 (c.1402+36T > C), IVS12+72 (c.1727T > G),3'UTR+44 (c.1962+44G > C)] and coding [p.A8A (c.24 A > C), p.A20A (c.60G > A); p.H33Q (c.99 T > G), p.Q433Q (c.99T > G), p.R105Q (c.314G > A); p.N181N (c.543 T > C); p.A314A (c.942G > C); p.A361 (Tc.1081G > A; p.T388T (c.116 G > C); p.V454I (c.1360G > A); p.R489R (c.1467C > T)] polymorphisms/sequence variants on IDUA expression in the Hurler patients from the Tunisian families is unknown [25].

Our study showed a heterogeneous pattern of mutations and polymorphisms among MPS I Tunisian patients.

The results of recent studies support the evidence of mutational heterogeneity of the *IDUA*, *IDS *and *GALNS *genes in patients with MPS I [25], MPS II [26] and MPS IVA [27] respectively. These MPS patients have different clinical presentation ranging from severe to mild. Since most patients have unique mutations, a comprehensive genotype-phenotype correlation is not feasible. At any rate, even in presence of recurrent mutations, a correlation has proved inapplicable. For example, the missense p.P533R mutation was usually reported associated with a severe phenotype [5], whereas this lesion identified on the contrary in our patients was found associated with mild and severe forms of the disease. This type of observation implicates others modifying or non genetic factor in the clinical presentation of disease.

A more comprehensive analysis of MPS I patients is essential. This must comprise not only a molecular screening for other known mutations, but also a complete screening for new mutations. In addition, a comprehensive clinical and epidemiological investigation could help assign the ethnic background of these patients.

## Conclusion

In summary, two novel mutations were identified in six MPS I patients from six Tunisian families. The severe missense mutation p.F177S was detected in one family and presumably resulted in misfolding and degradation of the mutant glycopepide. The other lesion was a severe small insertion 1587_1588 insGC that predicts premature termination of the IDUA protein. The identification of these mutations could provide genotype/phenotype correlations for the severe MPS I subtypes, accurate carrier detection, and prenatal diagnosis for MPS I families.

## Consent

Written informed consent was obtained from the patient for publication of this case report and accompanying images. A copy of the written consent is available for review by the Editor-in-Chief of this journal.

## Abbreviations

MPS I: Mucopolysaccharidosis I; IDUA: Alpha L iduronidase; PCR: polymerase chain reaction; MPS II: Mucopolysaccharidosis II; MPS IVA: mucopolysaccharidosis IVA; IDS: Iduronate -2-sulfatase; GALNS: N-acetylgalactosamine-6-sulfate-sulfatase.

## Competing interests

The authors declare that they have no competing interests.

## Authors' contributions

LC wrote the manuscript   LC, SK and RT performed all the work (PCR, sequencing...) in the laboratory.   HC, HB and MF analysed of the results  RF and ChVS participated in data analysis.   SF, SL and AM revised the manuscript and save final approval of the version to be published.   
